# 24-h Movement Guidelines and Overweight and Obesity Indicators in Toddlers, Children and Adolescents: A Systematic Review and Meta-Analysis

**DOI:** 10.1186/s40798-023-00569-5

**Published:** 2023-05-15

**Authors:** Adilson Marques, Rodrigo Ramirez-Campillo, Élvio R. Gouveia, Gérson Ferrari, Riki Tesler, Priscila Marconcin, Vânia Loureiro, Miguel Peralta, Hugo Sarmento

**Affiliations:** 1 CIPER, Faculdade de Motricidade Humana, Universidade de Lisboa, Estrada da Costa, 1499-002 Cruz Quebrada, Lisbon, Portugal; 2 ISAMB, Universidade de Lisboa, Lisbon, Portugal; 3 Exercise and Rehabilitation Sciences Laboratory, Faculty of Rehabilitation Sciences, Universidad Andres Bello, Santiago, Chile; 4 Department of Physical Education and Sport, University of Madeira, Funchal, Portugal; 5 LARSYS, Interactive Technologies Institute, Funchal, Portugal; 6 Escuela de Ciencias de la Actividad Física, el Deporte y la Salud, Universidad de Santiago de Chile (USACH), Santiago, Chile; 7 School of Health Sciences, Ariel University, Ariel, Israel; 8 KinesioLab Research Unit in Human Movement Analysis, Instituto Piaget, Almada, Portugal; 9 Department of Arts, Humanities and Sports, School of Education, Polytechnic Institute of Beja, Beja, Portugal; 10 Research Unit for Sport and Physical Activity, Faculty of Sport Sciences and Physical Education, University of Coimbra, Coimbra, Portugal

**Keywords:** Physical activity, Screen time, Sleep, Excess weight

## Abstract

**Background:**

Engaging in physical activity increases energy expenditure, reducing total body fat. Time spent in sedentary behaviours is associated with overweight and obesity, and adequate sleep duration is associated with improved body composition. This systematic review aimed to analyse the relationship between compliance with the 24-h movement guidelines and obesity indicators in toddlers, children and adolescents.

**Methods:**

A systematic review and meta-analysis was conducted. PubMed, Web of Science and Scopus were searched from inception to December 2021. Cross-sectional and prospective studies that analysed the relationship between 24-h movement guidelines and overweight and obesity written in English, French, Portuguese or Spanish were included. PROSPERO registration number is CRD42022298316.

**Results:**

The associations between meeting the 24-h movement guidelines and standardised body mass index were null in the two studies for toddlers. Seven studies analysed the relationship between compliance with the 24-h movement guidelines and overweight and obesity among preschool children. Of these seven studies, six found no association between compliance with 24-h movement guidelines and body composition. Among children and adolescents, 15 articles were analysed. Of these 15 studies, in seven, it was found that children and adolescents who meet the 24-h movement guidelines were more likely to have lower risks of overweight and obesity. The meta-analysis yielded a pooled OR = 0.80 (95% CI = 0.68 to 0.95, *p* = 0.012, *I*^2^ = 70.5%) in favour of compliant participants. Regarding participants’ age groups, compliance with 24-h movement guidelines seems to exert greater benefits on overweight and obesity indicators among children-adolescents (OR = 0.62, *p* = 0.008) compared to participants at preschool (OR = 1.00, *p* = 0.931) and toddlers (OR = 0.91, *p* = 0.853).

**Conclusion:**

Most included studies have not observed a significant relationship between compliance with the 24-h movement guidelines and overweight and obesity in toddlers, children and adolescents.

**Supplementary Information:**

The online version contains supplementary material available at 10.1186/s40798-023-00569-5.

## Key Points

- There are no associations between meeting 24-h movement guidelines and overweight and obesity for toddlers and preschool children.
- Among children and adolescents, the results are inconclusive. Some children and adolescents who met the 24-h movement guidelines were more likely to have lower risks of being overweight or obese.

## Introduction

The health benefits of engaging in physical activity, getting the recommended amount of sleep and reducing sedentary behaviour, mainly screen time, are well documented [1]. Several recommendations regarding physical activity, sedentary behaviour and sleep duration for toddlers, children and adolescents are available in the literature [1–4]. In 2016, the 24-h movement guidelines for children and youth aged 5–17 years were published in Canada for the first time [5]. One year later, guidelines were published for the early (0–4) years [6]. Briefly, these 24-h recommendations state that toddlers and preschoolers should accumulate at least three hours of physical activity (for preschoolers, at least one hour should be at moderate to vigorous intensity), ≤ 1 h/ day of screen time and 10 to13 hours of sleep [5, 6]. Children and adolescents should accumulate at least one hour of moderate-to-vigorous physical activity, ≤ 2 h of recreational screen time and 9–11 h of sleep (5–13 years old) or 8–10 h of sleep (14–17 years old) [5, 6]. These recommendations are an advance on existing guidance for physical activity [1, 2, 7], screen time [3] and sleep duration [4] because they integrate three different behaviours in a single recommendation. Its acceptance in the scientific and professional community occurred quickly and it began to be adopted in several countries and regions, such as Australia, New Zealand, South Africa and the Pacific region [8].

One of the health benefits of children and adolescents engaging in physical activity is preventing or treating obesity [1, 9]. Daily physical activity increases energy expenditure, contributing to a negative caloric balance and naturally reducing total body fat [10]. On the other hand, time spent in sedentary behaviours, mainly screen time, is associated with overweight and obesity [10, 11]. More time spent on screens is associated with more meal episodes, usually high-calorie snacks, which can explain the relationship between screen time and overweight and obesity [12]. Reducing screen time is also a strategy to prevent overweight and obesity. In addition, adequate sleep duration is associated with improved body composition [13, 14]. Children and adolescents who sleep less than recommended are more likely to be obese [14]. This means that sleep time must be respected as a physiological need of the body and as a behaviour that prevents various mental [15] and metabolic diseases [16, 17].

Although there is some evidence that physical activity, screen time and sleep duration are individually related to overweight and obesity [17–21], there is still discussion about how these behaviours interact and impact overweight and obesity in different age groups. Over the last decade, researchers have begun investigating the simultaneous associations of these three behaviours with health indicators [17, 22–24]. A previous review concluded that meeting 24-h movement guidelines has implications for health [25]. Therefore, the present systematic review aimed to evaluate the results of published studies that analyse the relationship between compliance with the 24-h movement guidelines [5] and overweight and obesity in toddlers, children and adolescents.

## Methods

### Registration

Before starting, the review protocol was registered in the International Prospective Register of Ongoing Systematic Reviews (PROSPERO) with ID number CRD42022298316. The developed protocol follows the standards for reporting systematic reviews and meta-analyses [26].

### Eligibility Criteria

The population, interventions, comparisons, outcomes and study design (PICOS) [27] framework was used to identify the studies following the objectives of the systematic review. Articles were required to analyse the relationship between 24-h movement guidelines and overweight and obesity. The following eligibility criteria were applied:

#### Population

It was expected to identify studies involving toddlers (1–4 years), children (5–12 years) and adolescents (13–17 years).

#### Exposure (Intervention)

Studies were included if they had reported the three behaviours of the Canadian 24-h movement guidelines for children and youth: physical activity, time spent in sedentary behaviour, specifically screen time, and sleep time [5]. For physical activity, studies were included if its assessment was self-reported, reported by parents or device based. For the assessment of screen time, the included studies could have assessed it by self-report or parent reporting for toddlers and younger children. The included studies would have to assess the duration of time spent sleeping by self-report, parent reporting or device based.

#### Comparison

According to the aim of the systematic review, potential studies did not have to be experimental or case–control. For this reason, the comparator or control group was not required for inclusion.

#### Outcomes

Overweight and obesity indicators were the primary outcomes. The outcomes could be body fat percentage using bioelectrical impedance analysis, body mass index z-scores, waist circumference and/or waist-to-height ratio or other objective measures of body composition assessment (e.g. using DEXA or plethysmography).

#### Study Design

Studies were observational or experimental to be included in the review. Case studies were excluded. Within these study designs, there were no further restrictions.

### Search Strategy

Articles published until 31 December 2022 were located by the authors using PubMed, Web of Science and Scopus databases. Keywords included combinations of: (obes* OR weight* OR overweight OR “body composition” OR fat* OR adiposity OR BMI OR body OR fitness*) AND (“24-Hour Movement” OR “Canadian guideline*” OR “physical activity guideline*” OR “physical activity recommendation*” OR “sedentary behaviour guideline*” OR “sedentary behavior guideline*” OR “sedentary behaviour recommendation*” OR “sedentary behavior recommendation*” OR “sleep guideline*” OR “sleep recommendation*” OR “screen time guideline*” OR “screen time recommendation*”). These terms were searched for the title and abstract of scientific articles. Additionally, the cross-referencing search was performed in the full-text read of potentially included articles.

To be included in the current review, articles were required to meet the following criteria: (1) analysed the relationship between 24-h movement guidelines and overweight and obesity and (2) written in English, French, Portuguese or Spanish languages. Articles from the grey literature were excluded.

### Study Selection

After searching each database, all studies were imported into Endnote 20, and duplicate records were eliminated. Two authors (AM and HS) analysed the titles and abstracts and selected the articles that would potentially enter the analysis. The same authors fully read all selected articles. There had to be a consensus for the articles to be included in the analysis. In the case of disagreement, an analysis was performed between the two authors (PM and EG), and a third author acted as moderator (VL). For an article to be excluded, it was necessary to present the reason.

### Data Extraction

After defining the articles that would enter the analysis, an Excel form was created for data extraction. From each article, information was extracted on authorship, publication year, the country where the study was carried out, the methodological design, the sample characteristics, the instruments and procedures for the assessment of overweight and obesity indicators (outcome), physical activity, screen time and sleep (exposures) and the main conclusions. In addition, information was extracted on the prevalence of toddlers, children and adolescents who complied with the total recommendation and the prevalence of those who did not comply with the recommendation in any of the behaviours.

### Methodological Quality Assessment

The methodological quality of the studies was assessed using the study quality assessment tool adapted from the National Institutes of Health/National Heart, Lung and Blood Institute (https://www.nhlbi.nih.gov/health-topics/study-quality-assessment-tools). The quality of each reviewed study was reported as poor, fair or good. The methodological quality of the articles was evaluated by one reviewer (HS) and confirmed by the larger review team. Disagreements were resolved by discussion among the team members.

### Quantitative Synthesis

The Comprehensive Meta-Analysis program (version 2; Biostat, Englewood, NJ, USA) was used. A minimum of three studies reporting the same outcome were required to perform the meta-analysis. The main outcome for the meta-analysis was overweight/obesity (e.g. BMI z-scores as a continuous outcome) relationship with 24-h movement guidelines compliance. Associations for weight status as a categorical outcome were not included.

To account for heterogeneity across studies, the weights of trials were proportional to their standard errors by applying an inverse variance using the DerSimonian and Laird random-effects model [28], computing meta-analyses reporting odd ratios (OR) with 95% confidence intervals (95% CIs). Heterogeneity was assessed using the *I*^2^ statistic, with values of < 25%, 25–75% and > 75% considered to represent low, moderate and high levels of heterogeneity, respectively [29].

## Results

### Literature Search

**Fig. 1:**
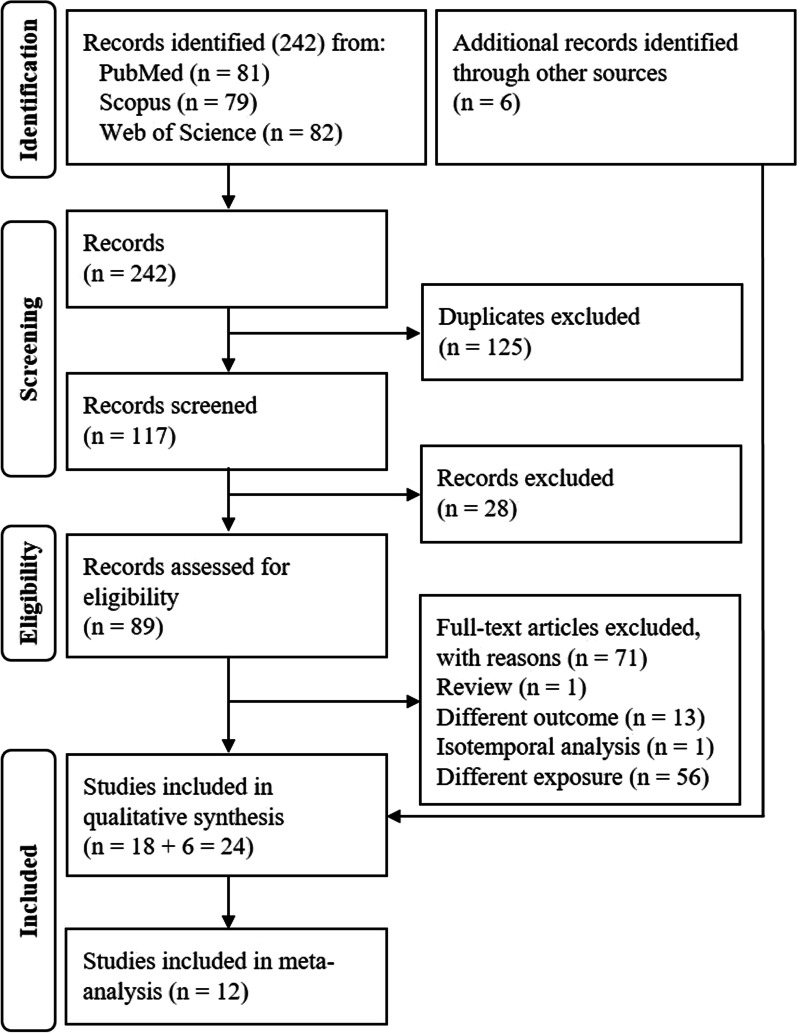
Flow diagram of study selection

A flow diagram of citations summarising the systematic review process is displayed in Fig. 1. The systematic literature search yielded a total of 242 potentially relevant publications. Of these records, 81 were identified in PubMed, 79 in Scopus and 82 in Web of Science. After excluding duplicates (*n* = 125), 117 publications were screened for inclusion in the review. A total of 28 articles were rejected at the title and abstract levels because they did not meet the inclusion criteria. Consequently, 89 potentially relevant citations were obtained. From these 89 articles, 71 were eliminated after a complete reading. Reasons for excluding articles were: review article (*n* = 1), different outcomes than overweight and obesity (*n* = 13), isotemporal analysis (*n* = 1) and not reporting a combination of physical activity, screen time and sleep duration (*n* = 56). Eighteen articles were identified as relevant. In addition to the 18 articles, six more were identified through the bibliography of consulted articles. In the end, the number of articles was 24.

### Study Characteristics

The study characteristics are summarised in Table 1. Of the 24 included articles, 20 were cross-sectional, two were prospective, one was a randomised controlled trial and one was cross-sectional and prospective. All studies were published in 2017 or later. The participants of two studies were toddlers [30, 31], seven studies were conducted among preschool children [13, 19, 32–36], and the other 15 included school-age children and adolescents. The number of study participants was 215,148 (353 toddlers, 6020 preschool children, 208,775 school-aged children and adolescents). The studies’ participants were from several different countries, two from Australia [31, 37], three from Canada [13, 30, 38], five from China (one from the mainland and two from Hong Kong) [18, 34, 39–41], one from the Czech Republic [42], one from Finland [35], two from Japan [19, 43], one from New Zealand [36], one from Spain [44], one from Sweden [32] and four from the USA [21, 45–47]. The remaining three studies analysed several countries: Belgium, Bulgaria, Germany, Greece, Poland and Spain [33]; Thailand, China, Malaysia, South Korea, Japan and Taiwan [48]; and Australia, Brazil, Canada, China, Colombia, Finland, India, Kenya, Portugal, South Africa, UK and USA [20].

**Table 1:** Study characteristics

Study	Sample (*n* and mean age or age range), study design, country	Obesity indicator	Movement behaviours (physical activity, sedentary behaviours and screen time and sleep)	Main findings
**Age group: toddlers**				
Lee et al. [30]	*n* = 151, 19.0 ± 1.9 months Cross-sectional Canada	BMI z-scores were calculated according to the WHO	PA was measured by accelerometer (ActiGraph wGT3X-BT). ST and sleep duration were reported by parents	The associations between meeting the specific and general combinations of the 24-h movement guidelines and BMI z-scores were null
Santos et al. [31]	*n* = 202 (98 boys, 104 girls), 19.74 ± 4.07 months Cross-sectional Australia	BMI z-scores were calculated according to the WHO	PA was measured by accelerometer (ActiGraph wGT3X-BT). ST and sleep duration were reported by parents	BMI was not associated with accomplishing any of the 24-h movement guidelines
**Age group: preschool children**				
Berglind et al. [32]	*n* = 830, 4–5 years Cross-sectional and prospective (1-year follow-up) Sweden	BMI z-scores were calculated according to the IOTF	PA was measured by accelerometer (ActiGraph GT3X +). ST and sleep duration were reported by parents	There was no cross-sectional or prospective association of the 24-h movement guidelines with BMI or BMI z-score at ages 4 and 5 years
Chaput et al. [13]	*n* = 803, 3.5 years Cross-sectional Canada	BMI z-scores were calculated according to the WHO	PA was measured by Actical accelerometers. ST and sleep duration were reported by parents	None of the combinations of recommendations was associated with adiposity
Decraene et al. [33]	*n* = 2468, 4.75 ± 0.43 years Cross-sectional Seven countries*	BMI z-scores were calculated according to the WHO	PA was measured by pedometers and accelerometer (GT1M, GT3X). ST and sleep duration were reported by parents	There was no association between guideline compliance with all three movement behaviours and adiposity
Feng et al. [34]	*n* = 173 (97 boys, 76 girls) Cross-sectional China (Hong Kong)	BMI z-scores were calculated according to the IOTF	PA was measured by accelerometer (activPAL). ST and sleep duration were reported by parents	No significant associations were found between meeting the guidelines and BMI
Kim et al. [19]	*n* = 421 (216 boys, 199 girls), 3–5 years Cross-sectional Japan	BMI z-scores were calculated according to the WHO	PA was measured by accelerometer (Active Style Pro HJA-750C). ST and sleep duration were reported by parents	Children who did not meet all the guidelines had a higher odds of being overweight/obese than those who met all guidelines
Leppänen et al. [35]	*n* = 778 (399 boys, 379 girls), 4.7 ± 0.9 years Cross-sectional Finland	BMI was calculated according to the IOTF. Trained researchers measured WC	PA was assessed 24 h/day over 7 days using ActiGraph wGT3X-BT accelerometer. ST and sleep duration were reported by parents	No significant associations were found between meeting 24-h movement guidelines and BMI or WC
Meredith-Jones et al. [36]	*n* = 547 (279 boys, 268 girls), 1–5 years RCT New Zealand	BMI z-scores were calculated according to the WHO. DXA scan measured body composition at 5 years of age	PA was measured by accelerometer (Actical) Parents reported ST duration Reported by their parents	Adherence to meeting all three guidelines at earlier ages was not related to BMI z-score or body composition at age 5, either cross-sectionally or prospectively
**Age group: children and adolescents**				
Chemtob et al. [38]	*n* = 630 (344 boys, 286 girls), 8–10 years *n* = 564 (313 boys, 251 girls), 10–12 years *n* = 377 (201 boys, 176 girls), 15–17 years Prospective Canada	BMI z-scores were calculated according to the WHO, % of body fat, waist circumference and waist-to-height ratio	PA was measured by accelerometer (GT3X, ActiGraph). Participants reported ST duration Sleep was reported by their parents and self-reported by adolescents	Children 8–10 years: compared with children who meet three components, children who meet two, one and no components had significantly higher adiposity Children 10–12 years: The adjusted model shows no significant association between meet guidelines and body composition prospectively (2-year changes) Adolescents 15–17: The adjusted model shows no significant association between meet guidelines and body composition prospectively (7-year changes)
Chen et al. [18]	*n* = 114,072 (56,103 boys, 57,969 girls), 13.75 ± 2.61 years Cross-sectional China	BMI z-scores were calculated according to the WHO	The HBSC survey questionnaire measured PA and ST. Measured by 1-item from the China Health and Nutrition Survey	Children and adolescents who met the 24-h movement guidelines were more likely to have lower risks of overweight and obesity
Haegele et al. [45]	*n* = 3582 (2367 boys, 1215 girls), 10–17 years Cross-sectional USA	BMI z-scores were calculated according to the WHO	PA, ST and sleep were self-reported	There was no relationship between accomplishing all 24-h movement guidelines and body composition
Hinkley et al. [37]	*n* = 471 (250 boys, 221 girls), 4.6 at baseline Prospective Australia	BMI z-scores were calculated according to the WHO, waist circumference	PA was measured by accelerometer (GT1M, ActiGraph). ST and sleep duration were reported by parents	No association was found between compliance with 24-h movement guidelines at baseline and BMI z-score 3 years later, but there was a significant inverse association between compliance and BMI z-score 6 years later
Hui et al. [48]	*n* = 12,590 (6563 boys, 6027 girls) Cross-sectional Six countries**	Body fat percentage was assessed using bioelectrical impedance analysis	PA was measured by IPAQ-SF. The Adolescents Sedentary Activity Questionnaire assessed ST. Sleep was self-reported	There was no relationship between accomplishing all 24-h movement guidelines and %BF
Jakubec et al. [42]	*n* = 679, 8–18 years Cross-sectional Czech Republic	BMI z-score, FM% and VAT were used as the adiposity indicators	PA was measured by accelerometer (ActiGraph GT3X +). ST was measured by a question from the HBSC study. Heuristic algorithm of the wake-up and fall-asleep times from the daily log was used to identify the sleep time	There was no association between 24-h movement guidelines and adiposity indicators
Katzmarzk and Staiano [46]	*n* = 357, 5–18 years Cross-sectional USA	BMI percentiles were computed according to CDC Growth Charts. WC, %BF, VAT and SAT were measured in a clinical setting	PA, ST and sleep were self-reported	Meeting more components of the 24-h guidelines was associated with lower levels of obesity and several cardiometabolic risk factors
Laurson et al. [21]	*n* = 674, 7–12 years Cross-sectional USA	BMI z-score and percentile were calculated according to CDC Growth Charts	PA was assessed by pedometer (Digiwalker SW-2000). ST and sleep were self-reported	Meeting the 24-h guidelines seems to have a protective effect against obesity
Roman-Viñas et al. [20]	*n* = 6128, 10.4 ± 0.6 Cross-sectional Twelve countries***	BMI z-scores were calculated according to the WHO	PA was objectively assessed using 24-h, waist-worn accelerometry. ST and sleep were self-reported	Meeting the 24-h movement guidelines was associated with lower OR for obesity
Shi et al. [39]	*n* = 1039, 11–18 years Cross-sectional China (Hong Kong)	BMI z-scores were calculated according to the WHO	PA was measured by accelerometer (activPAL). Sleep was measured by accelerometer (activPAL)	Overall, there were no differences in obesity levels between children and adolescents who comply and who do not comply with all recommendations
Suárez et al. [44]	*n* = 367, 7–11 years Cross-sectional Spain	BMI z-scores were calculated according to the IOTF. WC and waist-to-height ratio were calculated	PA and ST were self-reported Sleep was reported by their parents	Overall, there were no differences in obesity levels between children and adolescents who complied and who do not comply with the recommendations
Tanaka et al. [43]	*n* = 902, 9.4 ± 1.7, 6–12 years Cross-sectional Japan	BMI z-scores were calculated according to the WHO	PA was measured by accelerometer (Active stylePro HJA-350IT). ST and sleep were self-reported by children and their parents	Overall, there were no differences in obesity levels between children and adolescents who complied and who do not comply with the recommendations
Yang et al. [40]	*n* = 34,887 (18,074 boys, 16,816 girls), 11.4 ± 3.2 years Cross-sectional China	BMI z-scores were calculated according to the IOTF	PA, ST and sleep were reported by the adolescents and their parents together	Compared to those who met all three 24-h guidelines, those who met the sleep guideline were significantly associated with a higher risk of underweight and those who only met the MVPA or screen time guidelines were significantly associated with a higher risk of overweight or obesity
Zhou et al. [41]	*n* = 978 (520 boys, 458 girls), 9.1 ± 1.4 years Cross-sectional China	Chinese sex-specific and age-specific BMI cutoff points	PA, ST and sleep were self-reported, using items derived from the HBSC survey	Children who met more 24-h guidelines showed a lower risk of being overweight and obese and lower levels of %BF
Zhu et al. [47]	*n* = 30,478 (14,954 boys, 15,524 girls), 10–17 years Cross-sectional USA	BMI was calculated based on parent-reported height and weight	PA, ST and sleep were self-reported	Meeting physical activity guidelines was associated with the lowest odds of being overweight and obese

*BF* body fat; *BMI* body mass index; *CDC* Centers for Disease Control and Prevention; *FM* fat mass; *h* hour; *HBSC* Health Behaviour in School-aged Children; *IPAQ-SF* International Physical Activity Questionnaire—Short Form; *OR* odds ratio; *PA* physical activity; *SAT* subcutaneous adipose tissue; *ST* screen time; *VAT* visceral adipose tissue; *WC* waist circumference

^*^Belgium, Bulgaria, Germany, Greece, Poland, and Spain

^**^Thailand, China, Malaysia, South Korea, Japan, Taiwan

^***^Australia, Brazil, Canada, China, Colombia, Finland, India, Kenya, Portugal, South Africa, UK, USA

The outcome variable for overweight and obesity was the body mass index z-score in 20 studies. Only two studies used body fat percentage to assess overweight and obesity, one via bioelectrical impedance analysis [48] and one via dual-energy x-ray absorptiometry [36]. Accelerometers or pedometers were used to assess physical activity in two studies with toddlers [30, 31], seven studies with preschool children [13, 19, 32–36] and seven studies with children and adolescents [20, 21, 37–39, 42, 43]. Self-reported physical activity was used in eight studies with children and adolescents [18, 40, 41, 44–48]. Screen time was reported by parents in the studies with toddlers and preschool children and self-reported in studies with adolescents. Parents reported sleep time in studies with toddlers and preschool children. Among children and adolescents, sleep time was self-reported in most studies. Parents reported sleep time in two studies [43, 44], and in one, it was only objectively measured [39].

None of the studies was considered to be of weak methodological quality. Six studies with children and adolescents were of fair quality [18, 38, 40, 41, 44, 48], and the other 18 were of good quality (Additional files 1 and 2). Most studies did not report whether the outcome was blinded to the exposure status of participants.

### Main Findings

For toddlers, the associations between meeting the 24-h movement guidelines and body mass index z-score were null in both studies that analysed this relationship [30, 31].

Seven studies analysed the relationship between compliance with the 24-h movement guidelines and overweight and obesity among preschool children. Six studies found no association between meeting the 24-h movement guidelines and body mass index z-score [13, 32–36]. A non-significant association was also observed when body composition was assessed by waist circumference [35] or by dual-energy x-ray absorptiometry [36]. Only one study found that those who did not meet all the 24-h movement guidelines had higher odds of being overweight or obese than those who met the guidelines (OR = 1.14, 95% CI: 1.01, 1.29) [19].

Fifteen articles with school-age children and adolescents were analysed. Eight studies found that children and adolescents who met the 24-h movement guidelines were more likely to have lower risks of overweight and obesity [18, 20, 21, 37, 40, 41, 46, 47]. Six articles showed no differences in overweight and obesity levels in children and adolescents who comply and do not comply with all 24-h movement guidelines [39, 42–45, 48]. In one of the articles, the analyses were stratified by age, analysing children, early adolescents and adolescents [38]. The results were mixed, verifying a significant relationship between non-compliance with any recommendation and body mass index among children aged 8 to 10. However, no significant results were observed when analysing the relationship between non-compliance with any recommendation and body fat percentage.

**Fig. 2:**
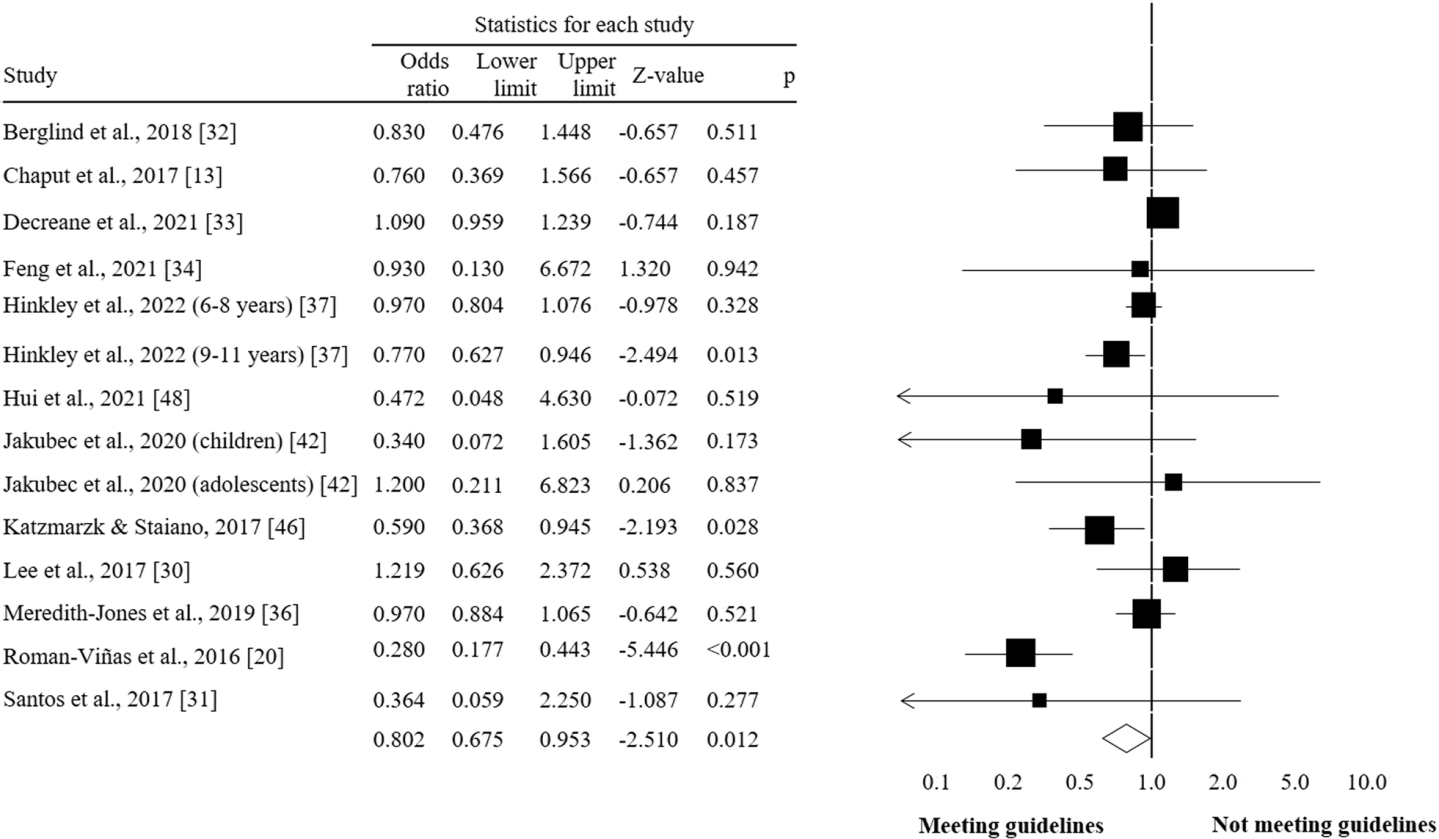
Forest plot of 24-h movement guidelines compliance and overweight and obesity indicators

The meta-analysis included 12 studies. Of 14 comparisons, 11 favoured compliant participants and three were statistically significant (*p* = 0.028 to < 0.001). The effects were fairly consistent, with the 95% CI for 11 out of 14 comparisons overlapping the mean, and a pooled OR = 0.80 (95% CI = 0.68 to 0.95, *p* = 0.012,* I*^2^ = 70.5%) (Fig. 2).

**Fig. 3:**
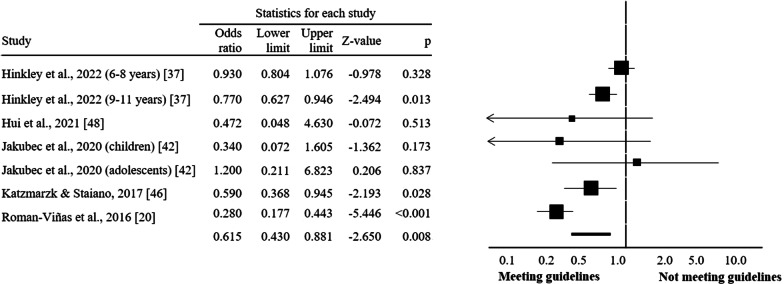
Forest plot of 24-h movement guidelines compliance and overweight and obesity indicators (children and adolescents)

**Fig. 4:**
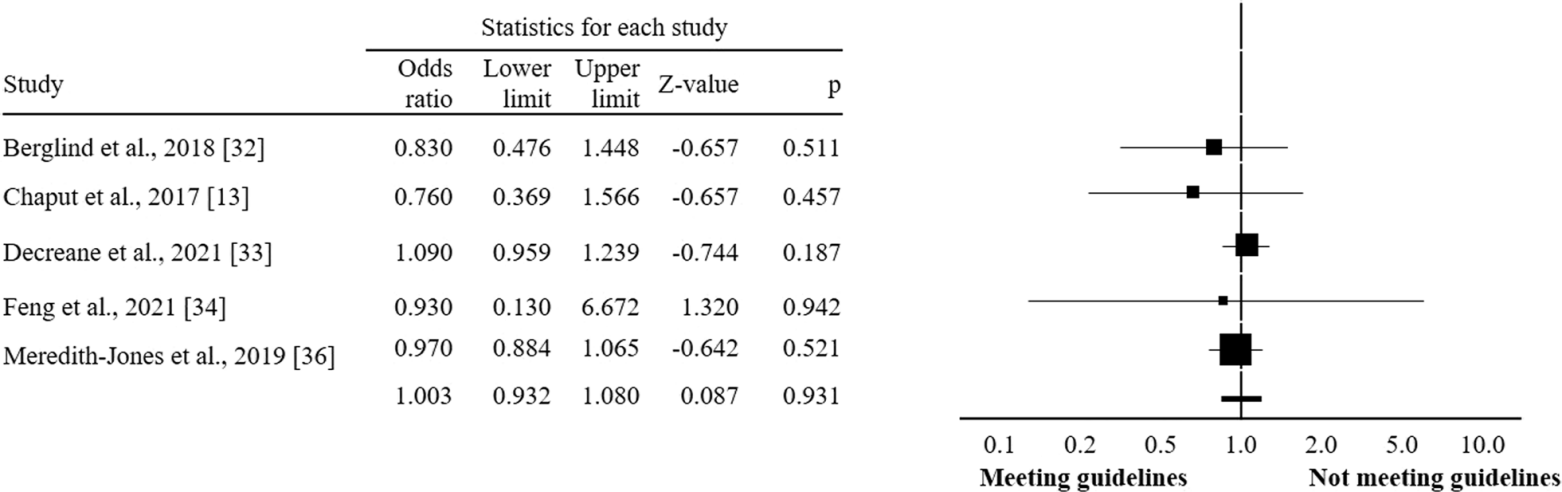
Forest plot of 24-h movement guidelines compliance and overweight and obesity indicators (preschoolers)

**Fig. 5:**
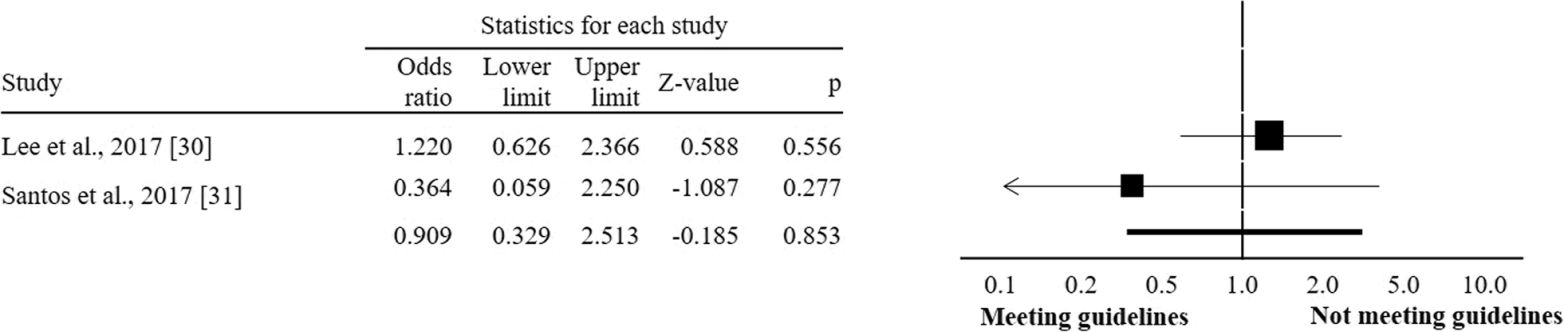
Forest plot of 24-h movement guidelines compliance and overweight and obesity indicators (toddlers)

Regarding participants’ age groups, compliance with guidelines seems to exert greater benefits on overweight and obesity indicators among children-adolescents (OR = 0.62, CI = 0.43 to 0.88, *I*^2^ = 78.3%; *p* = 0.008; Fig. 3) compared to participants at preschool (OR = 1.00, CI = 0.93–1.08, *I*^2^ = 0.0%; *p* = 0.931; Fig. 4) and toddlers (OR = 0.91, CI = 0.33 to 2.51, *I*^2^ = 33.0%; *p* = 0.853; Fig. 5).

## Discussion

This systematic review synthesised evidence from 24 articles examining the association between compliance with 24-h movement guidelines and overweight and obesity indicators among toddlers, preschool children, school-aged children and adolescents. Individually, physical activity, reduced screen time and sleep time seem to be related to obesity [11, 49]. As a result, it would be expected that the same would be observed when the three behaviours were combined. However, this was not the case in most studies. The findings indicate no associations between meeting 24-h movement guidelines and overweight and obesity for toddlers. However, only two articles were analysed, so care must be taken in interpreting these data. Generally, the results for preschool children showed no associations between meeting 24-h movement guidelines and being overweight or obese, except in one study [19]. For school-aged children and adolescents, the results were mixed. In most studies, no association was observed, but in eight studies, children and adolescents who met the 24-h movement guidelines were more likely to have lower risks of overweight and obesity [18, 20, 21, 37, 40, 41, 46, 47]. The meta-analysis indicated a favouring effect for guideline compliance, particularly among children and adolescents.

Neither individual movement nor combinations of behaviours were associated with overweight or obesity indicators for toddlers. Although these results are not theoretically expected, they align with previous studies [25]. Although most toddlers meet the physical activity recommendations [7], there were no significant relationships between physical activity and the body mass index in the two studies analysed [30, 31]. Furthermore, there were no significant relationships between full compliance with the 24-h movement guidelines and the body mass index. The main determinants of overweight and obesity for these ages are dietary factors (behavioural determinants) [50]. Thus, it is understandable that compliance with the 24-h movement guidelines may not significantly correlate with the body mass index.

Among preschool children, most studies did not indicate an association between compliance with the 24-h movement guidelines and overweight and obesity [13, 32–36]. However, one study did show that children who failed to meet the guidelines had a higher probability of obesity than those who met the guidelines [19]. In addition to this study, two others showed that compliance with certain individual recommendations was related to reducing adiposity [33, 35]. Nevertheless, the results are so different that a conclusion is unfeasible. For example, one study showed that meeting physical activity and sleep recommendations was associated with reducing adiposity [35]. Another study indicates that meeting the screen time recommendation alone reduces adiposity [33]. A possible explanation for these different results may be related to the fact that preschool children are very young and the impact of these behaviours on body composition is still not felt. Furthermore, recommendations for screen time may not be directly linked to overweight and obesity because the mere fact that a child is watching television, or using another device with a screen, does not increase total body fat. For this to happen, there must be another complementary action usually related to food intake. At younger ages, parents control food intake more [51], which may explain the absence of a significant relationship.

Individually, physical activity, reduced screen time and sleep time seem to be related to overweight and obesity in childhood and adolescence [11, 14, 49]. Nevertheless, for school-aged children and adolescents, the results were mixed. There was no association between compliance with the 24-h movement guidelines and overweight and obesity in most studies [38, 39, 42–45, 48]. In eight studies, children and adolescents who comply with the 24-h movement guidelines were less likely to be overweight or obese [18, 20, 21, 37, 40, 41, 46, 47]. In these studies, it was clear that physical activity was the behaviour that seemed to contribute most to the reduction of adiposity [20, 47]. These results align with previous investigations that sought to analyse the relationship between health indicators and the three behaviours that comprise the 24-h movement guidelines [23].

Mixed results, or the lack of a significant relationship between compliance with the 24-h movement guidelines and overweight and obesity, may be related to the fact that few toddlers, children and adolescents comply with the recommendations. One of the reasons why most toddlers, children and adolescents do not comply with the 24-h movement recommendations is the low prevalence of meeting the physical activity guidelines [52, 53]. Children and adolescents who meet the recommendations for physical activity improve cardiorespiratory fitness, musculoskeletal fitness, cardiometabolic health, motor skills, cognitive functions and academic outcomes and are less likely to experience depression [1, 9]. Although there are favourable associations between physical activity and reductions in overweight and obesity, the results are mixed [54]. It seems that going beyond the recommended physical activity levels is needed to affect adiposity in children. Isotemporal analyses showed that reducing sedentary time and increasing physical activity can reduce adiposity [55].

We acknowledged some limitations of this systematic review. First, most studies included were cross-sectional. This study design precluded conclusions regarding causality between movement behaviours and weight status or obesity. Second, in most studies, parents or teens reported sleep time. This information is based on when they went to bed and woke up. It is impossible to guarantee that toddlers, children and adolescents slept for all this reported time. Third, screen time was also reported, as there is not yet an instrument capable of measuring this time objectively. For teenagers, time on cell phone screens may not have been quantified. Fourth, several studies did not use representative population samples for the ages studied, making it difficult to generalise the results. Five studies not written in the English, French, Portuguese or Spanish languages were not included. For an in-depth understanding of these relationships, future studies may also benefit from including dietary behaviours and recommendations in their analysis.

## Conclusion

Most included studies have not observed a significant relationship between compliance with the 24-h movement guidelines and overweight and obesity in toddlers, children and adolescents. This is more evident in toddlers and preschool children. Two elements are critically needed to understand better the relationship between compliance with the 24-h movement guidelines and overweight and obesity: future longitudinal research design and the integration of more accurate methodologies to assess screen time and sleep time (including technological sensors).

## Supplementary Information

**Additional file 1: Table S1.** Canadian guidelines according to age groups.

**Additional file 2: Table S2.** Studies bias assessment using the NIH quality assessment tool for observational cohort and cross-sectional studies.

## Data Availability

Data sharing is not applicable to this article as no datasets were generated or analysed during the current study.
