# Supplementary material for: 24-h Movement Guidelines and Overweight and Obesity Indicators in Toddlers, Children and Adolescents: A Systematic Review and Meta-Analysis
Source: Sports Med Open. 2023 May 15;9:30. doi: 10.1186/s40798-023-00569-5 (PMC10185721; doi:10.1186/s40798-023-00569-5)
Supplement: Supplementary file 1 — Additional file 1: Table S1. Canadian guidelines according to age groups. [file 40798_2023_569_MOESM1_ESM.docx]

Table S1. Canadian guidelines according to age groups.

| **Movement behaviours** | **Toddlers (0-4)** | **Children**  **(preschoolers)** | **Children and adolescents** |
| --- | --- | --- | --- |
| **Physical activity** | ≥ 3 hours minutes per day | ≥ 3 hours minutes per day | ≥ 1 hour of moderate to vigorous intensity |
| **Screen time** | Not recommended (<1 year)  < 1 hour (1-2 years) | < 1 hour | < 2 minutes |
| **Sleep time** | 11 to 14 hours of good-quality sleep | 10 to 13 hours of good-quality sleep | 9 to 11 hours (5-13 years)  8 to 10 hours (14-17 years) |

https://csepguidelines.ca/
