# Supplementary material for: 24-h Movement Guidelines and Overweight and Obesity Indicators in Toddlers, Children and Adolescents: A Systematic Review and Meta-Analysis
Source: Sports Med Open. 2023 May 15;9:30. doi: 10.1186/s40798-023-00569-5 (PMC10185721; doi:10.1186/s40798-023-00569-5)
Supplement: Supplementary file 2 — Additional file 2: Table S2. Studies bias assessment using the NIH quality assessment tool for observational cohort and cross-sectional studies. [file 40798_2023_569_MOESM2_ESM.docx]

Table S2. Studies bias assessment using the NIH quality assessment tool for observational cohort and cross-sectional studies.

| **Reference** | **1** | **2** | **3** | **4** | **5** | **6** | **7** | **8** | **9** | **10** | **11** | **12** | **13** | **14** | **Overall** |
| --- | --- | --- | --- | --- | --- | --- | --- | --- | --- | --- | --- | --- | --- | --- | --- |
| **Age-group: toddlers** | | | | | | | | | | | | | | | |
| Lee et al., 2017 [30] | 1 | 1 | 1 | 1 | 0 | 1 | 1 | 1 | 1 | 1 | 1 | NR | 0 | 1 | Good |
| Santos et al., 2017 [31] | 1 | 1 | 1 | 1 | 0 | 1 | 1 | 1 | 1 | 1 | 1 | NR | 1 | 1 | Good |
| **Age-group: preschool children** | | | | | | | | | | | | | | | |
| Berglind et al., 2018 [32] | 1 | 1 | 0 | 1 | 0 | 1 | 1 | 1 | 1 | 1 | 1 | NR | NA | 1 | Good |
| Chaput et al., 2017 [13] | 1 | 1 | 1 | 1 | 0 | 1 | 1 | 1 | 1 | 1 | 1 | NR | 0 | 1 | Good |
| Decraene et al., 2021 [33] | 1 | 1 | 1 | 1 | 0 | 1 | 1 | 1 | 1 | 1 | 1 | NR | NA | 1 | Good |
| Feng et al., 2021 [34] | 1 | 1 | 1 | 1 | 0 | 1 | 1 | 1 | 1 | 1 | 1 | NR | 0 | 1 | Good |
| Kim et al., 2020 [19] | 1 | 1 | 1 | 1 | 0 | 1 | 1 | 1 | 1 | 1 | 1 | NR | 1 | 1 | Good |
| Leppänen et al., 2019 [35] | 1 | 1 | 0 | 1 | 0 | 1 | 1 | 1 | 1 | 1 | 1 | NR | NA | 1 | Good |
| Meredith-Jone et al., 2019 [36] | 1 | 1 | 1 | 1 | 0 | 1 | 1 | 1 | 1 | 1 | 1 | 1 | 0 | 1 | Good |
| **Age-group: children and adolescents** | | | | | | | | | | | | | | | |
| Chemtob et al., 2020 [38] | 1 | 1 | 0 | 1 | 0 | 1 | 1 | 1 | 1 | 1 | 1 | NR | 0 | 1 | Fair |
| Chen et al., 2021 [18] | 1 | 1 | 1 | 1 | 0 | 1 | 1 | NA | 1 | NA | 1 | NR | 1 | 1 | Fair |
| Haegele et al. 2021 [45] | 1 | 1 | 1 | 1 | 0 | 1 | 1 | 1 | 1 | 1 | 1 | NR | 0 | 1 | Good |
| Hinkley et al., 2020 [37] | 1 | 1 | 1 | 1 | 0 | 1 | 1 | 1 | 1 | 1 | 1 | NR | 1 | 1 | Good |
| Hui et al., 2021 [48] | 1 | 1 | 1 | 1 | 0 | 1 | 1 | NA | 1 | NA | 1 | NR | NA | 1 | Fair |
| Jakubec et al., 2020 [42] | 1 | 1 | 1 | 1 | 0 | 1 | 1 | 1 | 1 | 1 | 1 | NR | 0 | 1 | Good |
| Katzmarzk & Staiano, 2017 [46] | 1 | 1 | 1 | 1 | 0 | 1 | 1 | 1 | 1 | 1 | 1 | NR | 0 | 1 | Good |
| Laurson et al. 2014 [21] | 1 | 1 | 1 | 1 | 0 | 1 | 1 | 1 | 1 | 1 | 1 | NR | 0 | 1 | Good |
| Roman-Viñas et al., 2016 [20] | 1 | 1 | 1 | 1 | 0 | 1 | 1 | 1 | 1 | 1 | 1 | NR | 1 | 1 | Good |
| Shi et al., 2020 [39] | 1 | 1 | 1 | 1 | 0 | 1 | 1 | 1 | 1 | 1 | 1 | NR | 0 | 1 | Good |
| Suárez et al., 2021 [44] | 1 | 1 | 1 | 1 | 0 | 1 | 1 | NA | 1 | NA | 1 | NR | NA | 1 | Fair |
| Tanaka et al., 2020 [43] | 1 | 1 | 1 | 1 | 0 | 1 | 1 | 1 | 1 | 1 | 1 | NR | 0 | 1 | Good |
| Yang et al., 2022 [40] | 1 | 1 | 1 | 1 | 0 | 1 | 1 | NA | 1 | 0 | 1 | NR | 0 | 1 | Fair |
| Zhou et el. 2022 [41] | 1 | 1 | 1 | 1 | 0 | 1 | 1 | NA | 1 | 0 | 1 | NR | 0 | 1 | Fair |
| Zhu et al. 2020 [47] | 1 | 1 | 1 | 1 | 0 | 1 | 1 | 1 | 1 | 1 | 1 | NR | 0 | 1 | Good |
| Quality was rated as poor (0–4 out of 14 questions), fair (5–10 out of 14 questions), good (11–14 out of 14 questions)  Abbreviations: NA, not applicable; NR, not reported. | | | | | | | | | | | | | | | |

National Heart, Lung and Blood Institute Home Page. Available online:

<https://www.nhlbi.nih.gov/health-topics/study-quality-assessment-tools>
